# Intensive care diaries reduce new onset post traumatic stress disorder following critical illness: a randomised, controlled trial

**DOI:** 10.1186/cc9260

**Published:** 2010-09-15

**Authors:** Christina Jones, Carl Bäckman, Maurizia Capuzzo, Ingrid Egerod, Hans Flaatten, Cristina Granja, Christian Rylander, Richard D Griffiths

**Affiliations:** 1 ICU, Whiston Hospital, Warrington Road, Prescot L35 5DR, UK; 2 Faculty of Health & Life Sciences, University of Liverpool, Duncan Building, Daulby Street, Liverpool L69 3GA, UK; 3 ICU, Vrinnevisjukhuset, 25 Gamla Övägen, 601 82 Norrköping, Sweden; 4 Section of Anaesthesia and Intensive Care Medicine, University Hospital of Ferrara, 203 Corso Giovecca, 44100 Ferrara, Italy; 5 Rigshospitalet Dept. 7331, Blegdamsvej 9, University of Copenhagen, Copenhagen DK-2100, Denmark; 6 ICU, Haukeland University Hospital, 65 Jonas Liesvei, 5021 Bergen, Norway; 7 ICU, Hospital Pedro Hispano, Rua Dr Eduardo Torres, 4454-509, Matosinhos, Portugal; 8 ICU, Sahlgrenska University Hospital, SE-413 45 Gothenburg, Sweden

## Abstract

**Introduction:**

Patients recovering from critical illness have been shown to be at risk of developing Post Traumatic Stress disorder (PTSD). This study was to evaluate whether a prospectively collected diary of a patient's intensive care unit (ICU) stay when used during convalescence following critical illness will reduce the development of new onset PTSD.

**Methods:**

Intensive care patients with an ICU stay of more than 72 hours were recruited to a randomised controlled trial examining the effect of a diary outlining the details of the patients ICU stay on the development of acute PTSD. The intervention patients received their ICU diary at 1 month following critical care discharge and the final assessment of the development of acute PTSD was made at 3 months.

**Results:**

352 patients were randomised to the study at 1 month. The incidence of new cases of PTSD was reduced in the intervention group compared to the control patients (5% versus 13%, *P *= 0.02).

**Conclusions:**

The provision of an ICU diary is effective in aiding psychological recovery and reducing the incidence of new PTSD.

**Trial registration:**

NCT00912613.

## Introduction

ICU survivors, even with differing presenting diagnoses, have been shown to face common psychological problems during their recovery, including anxiety, depression and post-traumatic stress disorder (PTSD). PTSD is a condition triggered by the experience of a traumatic event [1], which must be severe enough to cause powerful subjective responses such as intense fear, helplessness and horror. It is characterised by a range of symptoms such as re-experiencing the event (flashbacks), avoidance of situations that remind one of the event, a numbed reaction and symptoms of increased arousal. The incidence of PTSD varies across different studies and units [2], but few studies have used a well-recognised diagnostic tool. One study using such a tool showed that on average 1 in 10 patients, who stay more than 48 hours in the ICU, will have the diagnosis [3]. The full diagnosis of PTSD is made if the symptoms the individuals are experiencing make it difficult for them to function in domains of their normal life, such as relationships or work. This has a significant impact on their quality of life leading to social isolation, marital problems, unemployment and long-term health problems, such as asthma, arthritis, headache, ulcers and cardiovascular problems [4]. Research suggests that the earlier treatment is provided the less the likelihood of patients going on to develop chronic PTSD [5].

The fragmentary nature of memories for the time in critical care and the high proportion of delusional memories, such as nightmares and hallucinations, that are recalled afterwards make it difficult for patients to make sense of what has happened to them [6] and has been shown to be one of the precipitants of PTSD in this population [3,7,8]. These memories are frequently described by patients as very vivid, realistic and frightening and may even involve patients thinking that nurses or doctors tried to kill them [6].

The use of an ICU diary with photographs started as an aid to fill in the gaps in patients' memories. A number of studies have shown that ICU diaries are well accepted by patients and families [9,10]. However, research into their possible role in aiding psychological recovery following critical illness is limited. One small randomised study (n = 36), has shown reduced depression and anxiety in those patients receiving a diary [11]. In our observational study of the precipitants associated with PTSD we noticed lower levels of PTSD-related symptoms in those patients receiving a diary [12]. We hypothesised that a diary explaining what happened to the patient in the ICU might help them fill in the significant gaps they have in their memories and put any delusional memories into context and so aid psychological recovery. It is possible that the ICU diary allows patients to change how they think about their experience of the ICU by providing a coherent narrative that can then be assimilated into autobiographical memory by rereading the diary over the months after their ICU discharge and so reducing the distress felt. The guidelines from the UK National Institute for Health and Clinical Excellence [13] for the treatment of PTSD recommend cognitive behavioural therapy (CBT). Changing how clients think about their traumatic experience is one of the aims of CBT.

The primary aim of the study was to test whether the provision of an ICU diary reduces the occurrence of new cases of PTSD (excluding those patients with pre-existing PTSD), particularly where patients' recall delusional memories.

## Materials and methods

### Protocol

The study was conducted in six general district hospitals and six university hospitals in six European countries. All hospitals in the study had prior experience with ICU diaries. All the individual hospitals gained ethical approval from their local research ethics committees and the research was carried out in compliance with the Helsinki Declaration.

The inclusion criteria were that the patients had been in the ICU and ventilated. Patients were excluded if they: stayed in the ICU for less than 72 hours; were ventilated for less than 24 hours; were too confused to give informed consent (including severe traumatic brain injury); and had pre-existing psychotic illness such as schizophrenic and manic depression (a confounding factor for psychological recovery) or diagnosed PTSD. As our previous study had shown that patients may already have all the symptoms required for a diagnosis of PTSD prior to ICU admission without this being previously recognised [3], the study protocol planned for the data analysis to only include patients with a new diagnosis of PTSD, excluding those subsequently recognised to have had previous non-ICU-related chronic PTSD. We decided to include patients with previous history of minor psychological problems, such as anxiety and depression, because this factor has been demonstrated to be associated with an increased risk of developing PTSD [3]. The decision to include only patients staying on the ICU for 72 hours and longer was both a logistical one to ensure that a reasonable ICU diary was available for the patient and because the risk of developing PTSD has been shown to be greater the longer the period of sedation [3].

The average number of patients admitted per unit in the study per year was around 400, of whom only around 100 were anticipated to stay for 72 hours and longer and survive to be discharged home from hospital. A minimum of 264 patients were required assuming: (a) 10% of patients suffered from new-onset PTSD [3]; (b) a projected 10% loss or withdrawal rate; and (c) a significance level of 5% and a power of 90%; and (d) a 50% reduction in PTSD based on our previous non-randomised study [12].

### Treatment conditions

All patients had an ICU diary written for them while they were in critical care, which the healthcare staff wrote and the family contributed to if they felt they could. The diary was a daily record of the patients' ICU stay, written in everyday language and accompanied by photographs. To standardise the diaries as much as possible a set of guidelines were provided to each centre [see Additional file 1]. The workload of writing in a diary is minimal at only a few minutes per day, except for starting the diary, which takes longer as an introduction is needed to explain why the patient had come to the ICU. Each study unit had a diary group that took on the burden of this. The patients were recruited to the study on the general wards at about one week after discharge from the ICU. Memories of the ICU were assessed at one week post-ICU using the ICU Memory Tool (ICUMT) [13], because recall of delusional memories (e.g. hallucinations and nightmares) from ICU assessed at this time point has been previously shown to be associated with new-onset PTSD [3]. The patients were also asked about any previous psychological history or previous history of undergoing traumatic events at this time point and this was cross-referenced with their clinical records.

Current best practice recommends that intervention studies for PTSD should be undertaken only once an initial assessment of symptom levels has been taken so ensuring that the study groups were comparable [14]. Baseline PTSD-related symptoms were assessed at one month post-ICU, as per best practice recommendations [15], just prior to randomisation to study group using the Post-Traumatic Stress Syndrome 14 (PTSS-14) screening tool [16]. The PTSS-14 was used at this time point as it is short (5 to 10 minutes to complete), can be used easily in an outpatient setting or over the telephone and does not overtire patients who are still very weak. The patients were then randomised at one-month post-ICU discharge to either receive their diary as soon as they wanted (intervention) or after they completed the final follow-up questionnaires at three months (controls).

The diary containing photographs and hand written text was introduced to the patient by a research nurse or doctor who ensured that they understood its contents but did not give any advice on what to do with it. Most of the discussions took place in an outpatient setting in a hospital but a small number were in the patients' own home. In units where the travelling distance was too great for the patient to return to the hospital the discussion of the diary took place over the phone. The diary was sent by registered post to the patient with a letter telling them not to read it and a date and time when the nurse would phone them and go through it with them using a photocopy. No other post-hospital contact was made until returning to the hospital or a phone interview at three months for formal diagnostic testing.

### Outcome measures

All the tools used in the study have been used in the ICU population before [3] and are well validated [15], the RACHEL (Raising Awareness, after Critical illness, of adverse Health Events in the Long-term) research group rechecked the validity of all the tools in translation in a previous multi-national study [3]. The ICUMT consists of 14 items that investigates the recall of hospital, ICU admission and discharge. A checklist of memories is included to increase the recall of ICU and the patient confirms their presence or absence. Baseline PTSD-related symptoms were assessed using the PTSS-14 screening tool, which is a 14-item questionnaire that has been validated with ICU patients [15] with concurrent validity (r = 0.86) against the PTSD Diagnostic Scale (PDS). Receiver operator curve analysis suggested the highest levels of sensitivity (86%) and specificity (97%) for diagnosis of PTSD, with an optimum decision threshold of 45 points and this was used to stratify the patients for analysis of PTSD-related symptom change. At three-months post-ICU, the PDS [17] was performed as the endpoint of the study. The PTSS-14 screening tool was also repeated. The PDS was administered as a diagnostic interview with the patient and allowed standardisation of the diagnosis of PTSD [17]. Moreover, it identifies the traumatic event responsible for symptoms as well as the time of the beginning of symptoms. It is one of the few measures in the PTSD literature that assesses all criteria (A - F), including functional impairment [18]. It also allows assessment of the severity of the patients' symptoms for each of the three PTSD symptom clusters, that is, re-experiencing, avoidance and physiological arousal. The higher the scores in the separate categories then the more severe the symptoms are. In the original validation paper [17] PTSD patients had a mean score of 8.95 for re-experiencing, 13.63 for avoidance and 11.02 for arousal. The original validation study showed a sensitivity of 82% and a specificity of 76.7%, showing a good overall level of diagnostic agreement with a clinical interview method of diagnosing PTSD [17]. This tool was chosen to standardise the assessment of PTSD across all the centres rather than relying on possibly variable clinical assessment by 12 different psychologists. An alternative would have been to use a standardised clinical interview but this would have been prohibitive in terms of resources. All centres were visited by one of the authors prior to their starting patient recruitment to ensure the tools were all administered in a consistent way.

It was impractical to guarantee blinding of the allocation of the diary as patients would volunteer their use. In order to reduce bias and ensure blinding of the diagnosis of PTSD at the three-month follow-up the researchers were only trained to interview and administer the PDS but were not made aware of the scoring calculation or in what way each question contributed to the score and final diagnosis. This calculation was performed by computer only at final analysis before the study code was broken. No interim analysis was planned or stopping rules designed as the intervention was already in widespread use and felt to be safe.

All tools had been previously translated into Swedish, Norwegian and Italian and checked for accuracy by back translation [3]. They were also translated into Danish and Portuguese and checked by back translation. The control group were able to receive their diary once the three-month questionnaires were completed. Those patients assessed using the PDS to have pre-morbid chronic PTSD (symptoms for more than three months after a traumatic event) prior to admission to ICU (often not previously diagnosed or labelled as anxiety) were excluded from the final analysis of new PTSD. These patients were recognised as they reported symptoms from some years before the ICU admission and with a different precipitant. It is not feasible so soon after ICU discharge when recruitment to this study occurred to have reliably undertaken the detailed PDS assessment required to exclude these previously undiagnosed patients with pre-existing chronic PTSD.

All patients had their illness severity assessed using an acute physiology and chronic health evaluation (APACHE) II score calculated for the day of admission to ICU. Length of ICU stay, admission diagnosis group, age and gender were also recorded.

### Study time points

At one to two weeks after ICU discharge, the patients completed the ICUMT. At one month they completed the PTSS-14 and were then randomised to the study group. At three months the PTSS-14 was repeated and the PDS completed.

### Assignment

The study was a block randomised controlled trial of the impact of receiving an ICU diary at one month within the first three months of recovery. The primary efficacy outcome was the occurrence of new-onset PTSD at three months post-ICU discharge (excluding those patients meeting the diagnostic criteria of chronic PTSD where the causation and symptoms pre-date the admission to ICU by more than three months).

Patients were recruited to the study one week after ICU discharge, when still on the general wards. Patients were assigned to treatment or control group at one month using a closed, non-transparent envelope technique, randomised in blocks of six through computerised random number generation. Data was entered directly onto an excel database in each study site with the randomisation status coded. Once the study closed, the data quality was checked and the database then locked. Data analysis was performed blind using coded data and the code only broken once the analysis was complete.

### Statistics

The statistical analysis was performed by one of the authors (CJ; who was not involved in the recruitment or follow-up of the study patients) using SPSS for Windows, version 14.0 (SPSS Inc, Chicago, Illinois, USA). Questionnaire data were treated as ordinal and analysed using non-parametric statistics [19]. Non-parametric statistics were used with interval data, for example, age [20]. However, parametric statistics were used with ordinal data if the variances across groups were approximately the same. The Levene statistic tested for equality of group variances. Clinical descriptors were used for comparison between control and intervention groups to ensure adequate randomisation. T tests and Mann Whitney U tests were employed when comparing two groups and univariate analysis of variance (ANOVA) using the F statistic to test group (>2) comparisons. The Scheffé *post hoc *test (set at *P*<0.05) was used for multiple comparisons. However, when the distribution was poor, the data were subjected to the distribution free equivalent method (Kruskal-Wallis one-way ANOVA for independent groups). Proportions were tested using Fisher's exact test. The PTSD outcome data are analysed based on new-onset PTSD after excluding those with established chronic PTSD.

## Results

### Participant flow and follow- up

**Figure 1:**
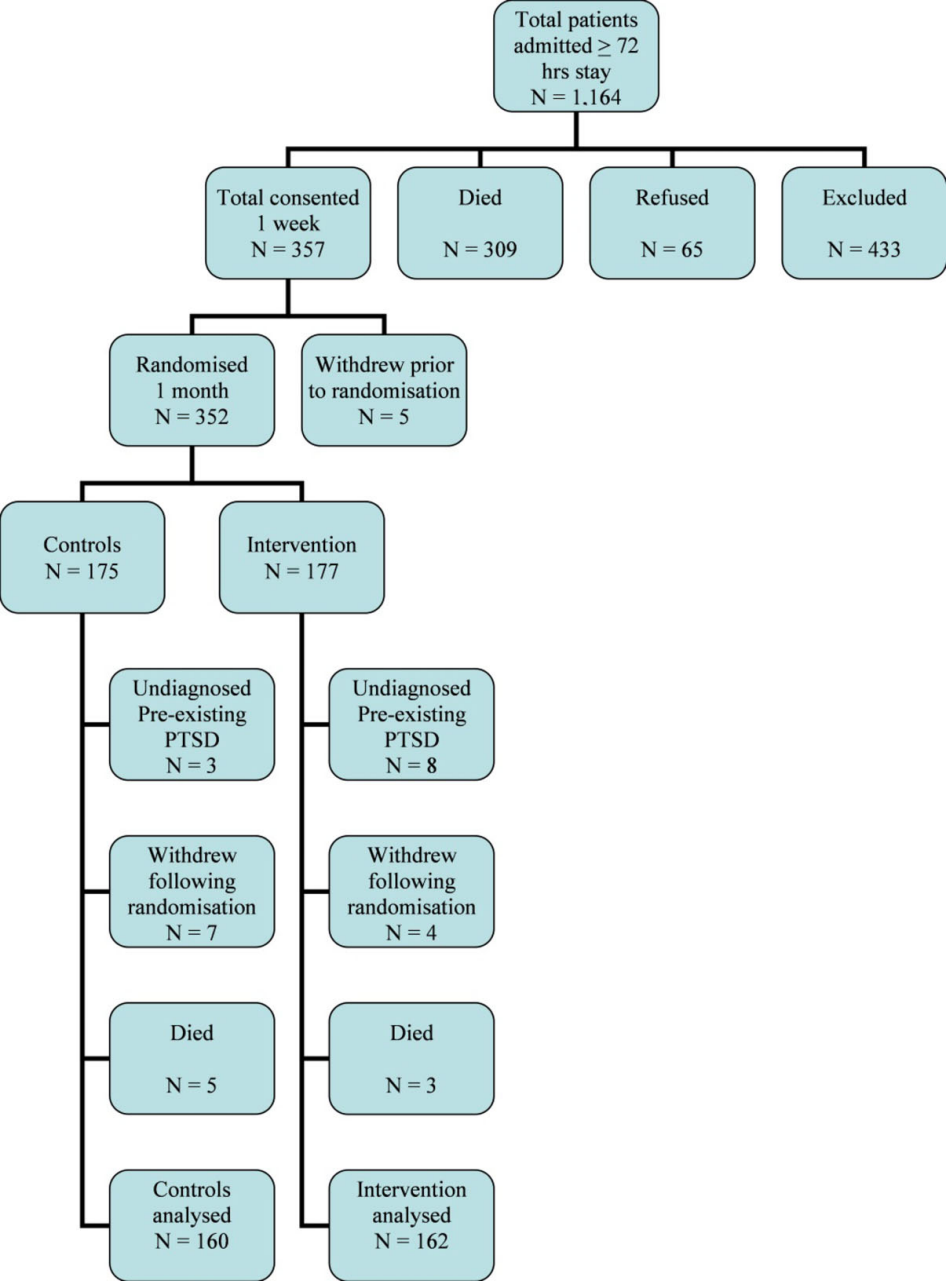
**CONSORT diagram of recruitment and follow-up**.

Recruitment at the 12 ICUs ran over a 12-month period between 2006 to 2008 with three months for follow-up, with a staggered start for each unit to ease training and supervision by the lead author (CJ). The Consort diagram (Figure 1) shows 1,164 eligible patients, 309 died in ICU or shortly after discharge, 65 refused to take part and 433 were excluded because of previous psychotic illness (n = 6), attempted suicide (n = 3), age younger than 16 years (n = 2), distance too great for follow-up (n = 10), transferred to another ICU (n = 274) or unresolving confusion at one to two weeks post-ICU admission (n = 138). At one-month post-discharge from ICU, 352 patients were randomised to the study and, of these patients, 333 completed their three-month follow-up questionnaires (see CONSORT diagram Figure 1). Three control and eight intervention patients were found to have previously undiagnosed pre-existing chronic PTSD using the PDS. Only two of these patients rated their ICU stay as being one of the traumatic events in their lives on the PDS (one control and one intervention) and all named an earlier event as the most traumatic event and had had PTSD symptoms for three to five years prior to their ICU admission. Five controls and three intervention patients died between the one and three-month follow-up, and seven controls and four intervention patients withdrew from the study. There was no difference in the one-month PTSS-14 between those who completed the study and those who dropped out (median PTSS-14 score 24 completed vs 28 for drop-outs, Mann-Whitney U *P *= 0.34). Of 177 intervention patients, 162 (91.5%) and of 175 controls 160 (91.4%) completed the three-month follow-up questionnaires and did not have pre-existing chronic PTSD. No adverse events were encountered during the study period.

### Study group comparisons

Following randomisation, there was no difference between the two study groups for age, length of ICU stay, hours ventilated, APACHE II, history of previous psychological problems or traumatic experiences, recall of delusional memories at one week or PTSD-related symptom levels using the PTSS 14 score at one month. However, there were more female patients randomised to the control group, 71 (41%) versus 54 (31%) in the intervention group (Table 1). The incidence of PTSD when examined in the control group was not found to be related to gender (12% in both males and females, Fisher's exact test *P *= 0.92). 49% of the controls and 51% of the intervention patients reported delusional memories from their time in ICU. The PTSS-14 scores at one month were statistically higher (Mann Whitney U *P *= 0.013) in those patients recalling delusional memories.

**Table 1:** Comparison of patient characteristics across study groups

	1 month randomisation			3-month follow-up		
				
	Study group		*P *values	Study group		*P *values
Variables (median, range)	Control (n = 175)	Intervention (n = 177)		Control (n = 160)	Intervention (n = 162)	

Age	59 (18-82)	60 (18-81)	NS	59 (± 16)	60 (± 15.5)	NS
ICU stay (days)	13 (3-71)	13 (3-79)	NS	13 (± 11.6)	13.8 (± 12.7)	NS
Hours ventilated	240 (24-1200)	212 (24-1500)	NS	240 (± 242)	216 (± 233)	NS
APACHE II severity score	18 (2-39)	20 (5-46)	NS	19 (± 6.5)	20 (± 7.3)	NS
Total PTSS 14 score at 1 month	25 (13-65)	22.5 (14-84)	NS	24 (± 11.6)	24 (± 12.2)	NS

Variables (n, percentage)						

Previous psychological problems	51 (29%)	51 (29%)	NS	43 (27%)	41 (27%)	NS
Previous traumatic experiences	38 (22%)	32 (18%)	NS	34 (22%)	25 (16%)	NS
Recall of delusional memories	91 (52%)	96 (54%)	NS	81 (52%)	85 (55%)	NS
Emergency admission to ICU	163 (93%)	157 (89%)	NS	149 (93%)	143 (88%)	NS
Gender (Male/female)	104/71	123/54	0.045	100/60	110/52	NS

Diagnostic groups (n, percentage)						

Respiratory failure	41 (23%)	35 (20%)	NS	35 (23%)	27 (18%)	NS
Sepsis	33 (19%)	21 (12%)	NS	31 (20%)	19 (12%)	NS
Circulatory failure	22 (13%)	22 (12%)	NS	17 (11%)	19 (12%)	NS
Multi-organ failure	20 (11%)	30 (17%)	NS	18 (11%)	24 (16%)	NS
Trauma (Total)	24 (14%)	27 (15%)	NS	23 (15%)	25 (16%)	NS
Multiple trauma without head injury	19 (11%)	14 (8%)	NS	19 (11%)	13 (8%)	NS
Multiple trauma with head injury	2 (1%)	7 (4%)	NS	2 (1%)	7 (4%)	NS
Isolated head injury	2 (1%)	5 (3%)	NS	2 (1%)	4 (3%)	NS
Other trauma	1 (0.5%)	1 (0.5%)	NS	0 (0%)	1 (0.5%)	NS
Combined (pulmonary/circulatory)	18 (10%)	19 (11%)	NS	16 (10%)	19 (11%)	NS
Gastrointestinal failure	8 (5%)	14 (8%)	NS	7 (5%)	13 (8%)	NS
Neurological failure	6 (3%)	5 (3%)	NS	5 (3%)	4 (3%)	NS
Other reasons	3 (2%)	3 (2%)	NS	3 (2%)	1 (2%)	NS
Planned	0 (0%)	1 (0.5%)	NS	0 (0%)	1 (0.5%)	NS

APACHE, acute physiology and chronic health evaluation; NS, not statistically significant; PTSS, post-traumatic stress syndrome.

### Analysis of effect of diaries

When pre-existing chronic PTSD, withdrawals and death were excluded, fewer intervention patients, compared with controls, were diagnosed as having new-onset PTSD at three months, 8 of 162 (5%) versus 21 of 160 (13.1%) (chi-squared = 7.15, *P *= 0.02; Table 2). This is despite 70 of 162 (43.2%) intervention patients and 76 of 160 (47.5%) controls reporting on the PDS that they found their ICU experience traumatic and so fulfilled criterion A for PTSD. Of the intervention patients, 87% received their diary at randomisation with the rest receiving theirs over the next month, leaving most with two months to reread the diary and 5% with one month before the three-month follow-up.

**Table 2:** Results at three months by study group

	Study group		
		
Variables (number, percentage)	Control (n = 160)	Intervention (n = 162)	*P *value
New-onset PTSD	21 (13.1%)	8 (5%)	0.02*
ICU seen as traumatic (PDS)	76 (47.5%)	70 (43.2%)	0.36

*Chi-squared test. PDS, PTSD Diagnostic Scale; PTSD, post-traumatic stress disorder.

### Change in PTSD symptoms

**Figure 2:**
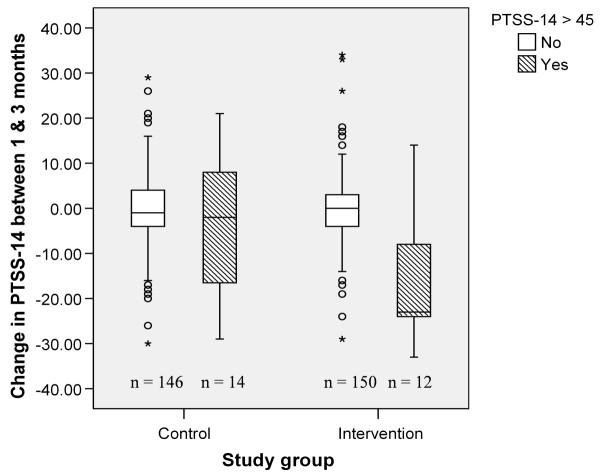
**Change in PTSS-14 scores between one and three months by study group and PTSS-14 of 45 or more at one month**. Patients in the intervention group with a post-traumatic stress syndrome (PTSS)-14 score above the cut-off of 45 at one month had a significant reduction in the PTSS-14 symptom score at three months (Fisher's exact test *P *= 0.04).

When the change in the PTSS-14 scores between one and three months was examined for all the patients completing the follow-up there was no overall difference between the controls and interventions (Mann Whitney U *P *= 0.737). However, in a *post-hoc *analysis, when the literature cut-off score of 45 or more [16] was applied, 12 intervention and 14 control patients scored in this range at one month. By the three-month follow-up there was a significant reduction in the PTSS-14 score for those scoring 45 or more at one month in the intervention group (Mann Whitney U test *P *= 0.04; Figure 2). The median change in the score for those intervention patients scoring very highly for PTSD-related symptoms between one and three months was -23, showing a significant reduction compared with the equivalent controls which was -2.

### Patients' comments

Patient feedback about the diaries was very positive with most of the intervention patients receiving the diary at the one-month follow-up and reading it a median of three times (0 to 20 range). Only one patient had not read the diary. Of the intervention patients, 148 (84%) said that others had read the diary, most commonly the family (100%), friends (36%), colleagues (5%) and healthcare staff (4%). When asked what the intervention patients felt helped most, only two (1.4%) patients mentioned the meeting with the nurse, 66 (49%) felt reading the text in the diary was most helpful, 49 (36%) thought the combination of photographs and text and 21 (15%) thought the photographs helped most.

## Discussion

Our hypothesis was that a diary explaining what happened to the patient in ICU might help patients fill in gaps in their memories, place any delusional memories into context and aid psychological recovery. The study showed that the provision of an ICU diary, outlining the patients' stay in ICU on a day to day basis, given at one month into the patients' recovery helps to reduce the incidence of new acute-onset PTSD reducing its impact on the patients' long-term quality of life [21]. ICU patients developing PTSD may be haunted by their memories and may try to suppress them; however, trying not to think about such emotionally charged memories leads to more unbidden thoughts accompanied by greater physiological arousal [22]. Learning to modulate their feelings and reduce the physiological arousal is the first step to recovery. CBT is recommended for the treatment of PTSD and a similar mechanism may be operating with the diaries because it helps patients to change how they think about their illness as they reread the story and build an autobiographical memory.

There was one slight difference between the study groups at randomisation; there were more females in the control group and at least one study has suggested that high levels of PTSD-related symptoms are more common in women [23]. However, this is not a consistent finding across studies [2] and in the control group gender was not related to the development of PTSD in this study.

Family members can suffer from PTSD themselves [24] and may also be helped by the diaries because it clarifies the story for them too and helps to facilitate communication with the patient about their treatment. In addition writing in the diary when the patient is in ICU may allow them to express some of their feelings. A strong relation has been found between high levels of PTSD-related symptoms in family members and those in patients [24] so a therapy, such as a diary, that is shared between the patient and the family may be better than one that just concentrates on the patient.

The mechanism behind the development of PTSD is still not clear. Some researchers have focused on noradrenergic activity as a key component of the stress response and tried to manipulate this to reduce the noradrenergic activity and the enhanced startle response, which is part of PTSD [25]. Other PTSD researchers have suggested that the helplessness experienced at the time of the trauma and the biological effects of being unable to escape lead to the profound changes seen in PTSD [26]. Ehlers and Clark's [27] PTSD model hypothesises that it is the quality of an individuals' cognitive processing during a traumatic event that is important in the development of chronic PTSD. Those individuals who report feeling confusion and overwhelmed as they experienced the traumatic situation are more likely to suffer from chronic PTSD. ICU patients' ability to process information is likely to be compromised by a number of factors, such as critical illness, delirium, sleep deprivation, sedative drugs and opiates. They are likely to be unable to process the meaning of the events happening to them and may instead experience delusional events such as hallucinations or nightmares and so be predisposed to high levels of chronic PTSD. A number of studies have examined the influence of giving a β-adrenergic receptor antagonist [28,29] or cortisol therapy [30-32] to try to reduce the incidence of PTSD; however, these therapies may be problematic in the critical care population and more research is needed to clarify their role.

Concern has been expressed by some that the diaries could be seen as a one-off debriefing session, which have been shown to increase the risk of PTSD [33]. However, as many patient's memories are fragmentary and much contained in the diary is new to the patient, this seems unlikely and is not supported by the results of this study.

Diaries are not without cost; there has to be a commitment from the staff to write something in the diary every day and take photographs when important changes happen. In addition an experienced nurse is needed to go through the diary with the patient to ensure that they understand its contents, but this is not significantly more than might have been provided by an unstructured discussion in the past. However, compared with providing formal CBT to all patients struggling to cope with their experience this is likely to be a cost-effective therapy.

The study had a number of strengths but also limitations and possibility of bias. One of its strengths is the low attrition rate, which can be attributed to the use of research staff with previous experience of following patients up and the direct interview style method rather than using postal questionnaires. In addition the wide recruitment across 12 ICUs facilitates the generalisability of the results. It was not possible to fully blind the receipt of the diary from the investigators because patients will often mention them. However, the scoring of the PDS is complicated and in components and is therefore difficult to influence unconsciously. The interviewing investigators were kept blind to the separate symptom group scores and the overall diagnosis of PTSD. In addition the intervention patients had a discussion session with an experienced investigator to go through the diary that could have influenced the results. However, the current practice for both control and study group patients at several of the study sites was to give them verbal information about their illness prior to discharge from hospital, which would have entailed a similar discussion. This could be examined in further studies. Another possible limitation is the fact that not all patients could get back to the hospital to receive their diary or for the final interview due to travelling distance and so these interviews were conducted by telephone. This was a pragmatic study and tried to mimic what would be normally done clinically. The majority of study participants were interviewed face to face in a hospital setting so the likelihood of this having an influence on the incidence of PTSD is not great. Finally, it would have been preferable to have used the PDS at one month so that patients with previous PTSD, not related to their ICU stay, were recognised prior to randomisation and excluded. The PDS can take one hour to complete and for some patients at one month this is still too long for them to maintain concentration and give reliable information. This would also have been difficult where the interview had to be conducted by telephone. A compromise was to make the diagnosis based on the three month PDS and exclude those patients with pre-existing PTSD from the analysis.

The patients in this study reported finding both the text and the photographs helpful in understanding their illness. This is a simple and very practical intervention, which this study shows is effective in reducing the incidence of new PTSD after critical illness. The UK NICE guidelines on the treatment of PTSD suggest that targeting at-risk groups rather than blanket interventions should be practiced [12]. This would mean that ICU patients would only receive a diary if they had high levels of symptoms. However diaries have been seen simply as a source of information for patients about their illness and it has been suggested that all patients staying on the ICU for more than 48 hours should have one [34]. In practice their use may fall somewhere in the middle with the longer-stay patients likely to benefit the most and being an achievable target. Those patients with low levels of PTSD symptoms may still be happy to receive the information it contains. The study shows that those intervention patients scoring very highly for PTSD symptoms using the PTSS-14 got the biggest reduction in symptoms by three months, whereas the majority of those not scoring highly remained unchanged. However, this was a *post-hoc *analysis resulting in a low number of patients for analysis. The fact that when all the patients were examined there was no significant difference between the two study groups in the change in PTSS-14 scores between one and three months is not surprising as only about 10% of the patients scored more than 45 on the PTSS-14 at one month and the 90% of patients with lower scores masked the result.

The lowest occurrence of new-onset PTSD in the intervention patients is an important first step in helping these patients to put their lives back together after critical illness.

## Conclusions

The provision of an ICU diary was associated with a reduction in the incidence of new-onset PTSD. Patient feedback about their diary was very positive with the majority reading it a number of times over the two-month follow-up period. The lower occurrence of new-onset PTSD in the intervention group is encouraging and suggests that an ICU diary may represent an important first step to help patients come to terms with their experiences.

## Key messages

• ICU diaries reduce new-onset PTSD following critical illness.

• Those patients with high levels of symptoms of PTSD at one month post-ICU benefit most from the diary in terms of reduction in new-onset PTSD.

• All the intervention patients were very positive about their diary.

• For the majority of the intervention, patients family, friends, work colleagues and healthcare professionals had also read the diary.

## Abbreviations

ANOVA: univariate analysis of variance; CBT: cognitive behavioural therapy; ICUMT: ICU Memory Tool; PDS: the PTSD Diagnostic Scale; PTSD: post traumatic stress disorder; PTSS-14: Post-Traumatic Stress Syndrome 14.

## Competing interests

The authors declare that they have no competing interests.

## Authors' contributions

CJ co-conceived the study, participated in its design and coordination, performed the statistical analysis and drafted the manuscript. CB developed the diary concept, coordinated the study in Sweden, collected data and helped to draft the manuscript. MC coordinated the study in Italy and helped to draft the manuscript. IE coordinated the study in Denmark and helped to draft the manuscript. HF coordinated the study in Norway and helped to draft the manuscript. CG coordinated the study in Portugal and helped to draft the manuscript. CR helped coordinate the study in Sweden and to draft the manuscript. RDG co-conceived the study and participated in its design and helped to draft the manuscript. All authors read and approved the final manuscript.

## Supplementary Material

Additional file 1 **Patient diary guidelines**. This contains the guidelines used in the study to ensure all study centres wrote diaries in a similar way. Click here for file
