# Supplementary material for: Intensive care diaries reduce new onset post traumatic stress disorder following critical illness: a randomised, controlled trial
Source: Crit Care. 2010 Sep 15;14(5):R168. doi: 10.1186/cc9260 (PMC3219263; doi:10.1186/cc9260)
Supplement: Additional file 1 — Patient diary guidelines. This contains the guidelines used in the study to ensure all study centres wrote diaries in a similar way. [file cc9260-S1.DOC]

**Additional file 1**

**PATIENT DIARY GUIDELINES**

**Introduction**

Patient diaries have been introduced for Intensive Care patients at Whiston Hospital as a way of helping patients to understand what has happened to them whilst they have been critically ill.

Psychological problems post critical illness are common (Griffiths et al 1996, Jones et al 2001). Patients may suffer from memory distortions, such as nightmares and hallucination, and periods of amnesia. Many patients have no memory of their Intensive Care stay at all (Jones 1998).

Bäckman and Walther (2001) describe positive results from using detailed narratives in diary format for Intensive Care patients. Studies have suggested that the diaries are beneficial for patients with little or no recall of their ICU stay or who have frightening delusional memories.

ICU Patient Diaries have been discussed and given backing from the Trust’s Ethics Committee, Legal Department and Caldicott Guardian; from these discussions it has been decided that the diaries are considered a therapy rather than a medical record.

This booklet provides guidelines on recording and storage of diaries. The contents may be revised as appropriate at any time.

**Starting a diary**

When a diary is commenced for a patient on ICU, please ensure that the patient’s name is added to the diary list in the Diary Register. This allows the diary team to keep track of who has diaries and where that diary is.

**Photographs**

Photographs may be taken of patients but they cannot be given to the family without their consent. Relatives may be photographed with the patient if they wish. Therefore photographs must not be entered into the diaries until the patient has seen those photographs and given their consent. **Photographs must not be given to the patient’s family or friends.**

An initial photograph is recommended when the patient is fully sedated and ventilated.

Subsequent photographs may be taken to show the patient’s progress, e.g. awake, sitting in a chair, or perhaps on dialysis for example.

A space should be left in the diary for the photograph to be mounted at a later date. The space should be labelled diagonally “photograph space” and the area surrounding it hatched out to avoid people writing in the space.

Once taken, the photographs must be stored immediately for safekeeping.

**Diary format and writing style**

The patient’s name, hospital number, bed space and date of admission to ICU should be written at the top of the first page for identification purposes. The outside of the diary can be labelled with an ID sticker which does **not** have the patients address on.

All entries must be made clearly, in black ink, dated and signed. The first entry should include a description of the reason for admission to ICU.

Avoid including information that could be of a sensitive nature, or that a patient may wish to keep confidential. Examples include malignancy, HIV status, sexuality or substance abuse. A sensible approach is to write only what you would be comfortable to disclose verbally to a patient or relative at the bedside.

Entries need not be made every shift. However entries should be made daily as much as possible and when there is a significant event or milestone to write about. Examples include extubation, a tracheostomy procedure, sitting out of bed for the first time. If progress is slow, still try to make regular entries. If a patient is restless write about this as they may remember hallucinations from this period.

Include the relatives. Give them an information sheet about the diaries. Encourage them to write to say that they have been visiting. They may wish to include what has happened at home or anything that the patient has a particular interest in.

All members of staff are invited and are welcome to make diary entries. A diary with contributions from nurses, doctors, physiotherapists, chaplains and relatives is likely to hold more meaning than a diary filled in by one person alone.

Avoid jargon and abbreviations. Use laymen’s terms when describing clinical terminology for the first time in the diary. Try to relate what you write to how you would normally verbalise the information to a patient or relative.

Writing style should always be professional and relevant. As much care and consideration should be taken with diary entries as any other form of professional documentation.
